# Comparative analysis of cis-regulation following stroke and seizures in subspaces of conserved eigensystems

**DOI:** 10.1186/1752-0509-4-86

**Published:** 2010-06-17

**Authors:** Michal Dabrowski, Norbert Dojer, Malgorzata Zawadzka, Jakub Mieczkowski, Bozena Kaminska

**Affiliations:** 1 Laboratory of Transcription Regulation, Department of Cell Biology, Nencki Institute, Pasteura 3, 02-093 Warsaw, Poland; 2 Institute of Informatics, Faculty of Mathematics, Informatics, and Mechanics, University of Warsaw, Banacha 2, 02-097 Warsaw, Poland

## Abstract

**Background:**

It is often desirable to separate effects of different regulators on gene expression, or to identify effects of the same regulator across several systems. Here, we focus on the rat brain following stroke or seizures, and demonstrate how the two tasks can be approached simultaneously.

**Results:**

We applied SVD to time-series gene expression datasets from the rat experimental models of stroke and seizures. We demonstrate conservation of two eigensystems, reflecting inflammation and/or apoptosis (eigensystem 2) and neuronal synaptic activity (eigensystem 3), between the stroke and seizures. We analyzed cis-regulation of gene expression in the subspaces of the conserved eigensystems. Bayesian networks analysis was performed separately for either experimental model, with cross-system validation of the highest-ranking features. In this way, we correctly re-discovered the role of AP1 in the regulation of apoptosis, and the involvement of Creb and Egr in the regulation of synaptic activity-related genes.

We identified a novel antagonistic effect of the motif recognized by the nuclear matrix attachment region-binding protein Satb1 on AP1-driven transcriptional activation, suggesting a link between chromatin loop structure and gene activation by AP1. The effects of motifs binding Satb1 and Creb on gene expression in brain conform to the assumption of the linear response model of gene regulation. Our data also suggest that numerous enhancers of neuronal-specific genes are important for their responsiveness to the synaptic activity.

**Conclusion:**

Eigensystems conserved between stroke and seizures separate effects of inflammation/apoptosis and neuronal synaptic activity, exerted by different transcription factors, on gene expression in rat brain.

## Background

Stroke and seizures-induced neurodegeneration share a number of biological processes, including increased neuronal activity, neuronal plasticity, inflammation, and apoptosis [1,2]. Separation of effects of these processes on gene expression, identification of participating transcription factors, and comparison of transcriptional regulation between the two pathological conditions remain a challenging task. Global gene expression following stroke and seizures were compared before at a single time-point [3], but no comparison of time-series gene profiling datasets from the two conditions was reported to date.

Alter et al. first introduced a concept that orthogonal components (eigensystems) resulting from the singular value decomposition (SVD) of time-series gene expression dataset [4,5] may help to separate concurrent effects of different processes and regulators on gene expression. These authors proposed that an eigen *array *may reflect a genome-wide input from a particular regulator, with the corresponding eigen *gene *reflecting this regulator's activity across the samples (arrays). For an illustration of the SVD nomenclature, when applied to gene expression - see Additional file 1.

A number of recent studies concentrated on usefulness of eigengenes [6-10], whereas the properties and interpretation of eigenarrays remained relatively less explored. We previously suggested that conservation of eigenarrays between related biological systems may identify eigensystems of biological origin [11]. In the same work, utilizing a comparative SVD approach we identified an eigensystem conserved between hippocampal development and differentiation of hippocampal neurons in vitro. Analysis of cis-regulation of that eigensystem revealed that it reflected exit of neural precursors from the cell cycle and beginning of neuronal differentiation, regulated by transcription factors E2f1 and Nr2f1 [12].

Bayesian Networks (BN) learning approach is a well-established method of modelling gene regulation and interactions between gene regulatory motifs, starting from gene expression data [13] or gene expression and genomic sequence data [14-20]. The use of linear regression in analysis of gene cis-regulation is grounded in the linear response model of gene regulation [21,22].

Here, we report a time-series dataset from gene expression profiling in the rat MCAO model of stroke, and compare these data to the published time-series dataset from the kainate-induced seizures model [23]. By comparative SVD approach, followed by Bayesian network analysis of cis-regulation, we identified two conserved eigensystems separating the effects of different well-defined biological processes on gene expression and regulated by distinct sets of transcription factor binding sites. The results obtained on either dataset were validated on the other.

## Results

### Experimental data and analysis setup

We compared two time-series gene expression datasets from experimental rat models of stroke and epilepsy, which were the transient middle-cerebral artery occlusion (MCAO) and the kainate-induced seizures, respectively. The MCAO dataset was generated in our laboratory and probed gene expression in the cortex of the ischemic hemisphere at four time-points (6, 12, 24, 48 h) following a 90 minutes occlusion of the right middle-cerebral artery in adult anesthetized rats, and included sham-operated animals as controls. The kainate dataset, published by Koh and co-workers [23] probed gene expression in the hippocampus of adult rats at five time-points (1, 6, 24, 72, 240 h) following the injection of kainate - a neurotransmitter analogue inducing seizures, which can last for several hours, followed by a seizure-free latent period.

As immobilization of a conscious animal and injection alters gene expression in the brain, this dataset included a control time-series following the injection of saline.

**Figure 1:**
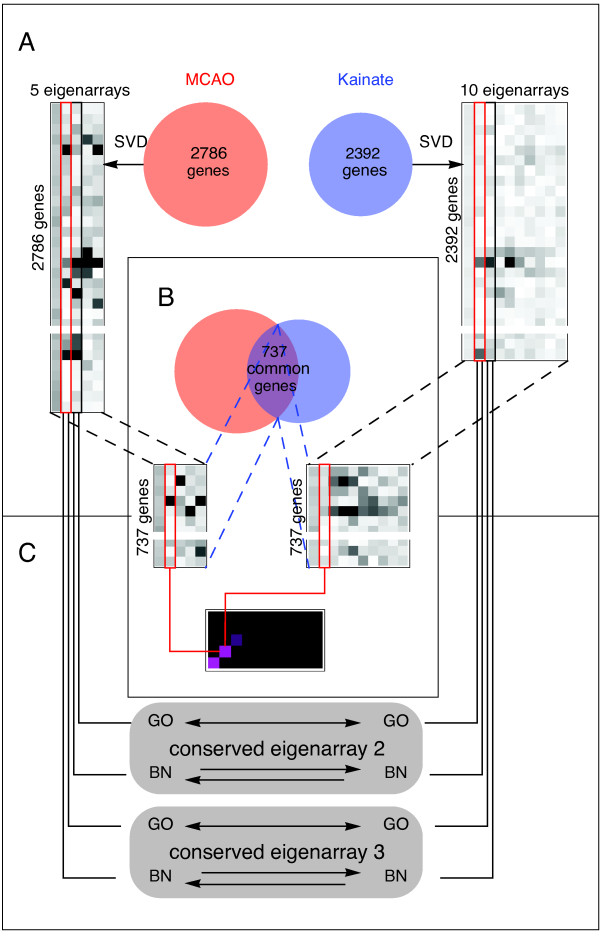
**Design of the study **(**A**) The datasets from gene profiling of rat brain following stroke in the MCAO model and kainate-induced seizures were each separately transformed by SVD. (**B**) The eigenarrays resulting from the SVD of either dataset were compared by correlation analysis performed for the genes common between the two datasets. (**C**) For the emerging conserved eigensystems 2 and 3, separately for all the genes in either dataset, we studied their functional Gene Ontology (GO) associations and employed Bayesian Networks (BN) to study their cis-regulation. The results obtained on one dataset were then compared (GO) or statistically tested (BN) on the other.

The overall design of our study is illustrated in Figure 1. We transform each dataset (MCAO, kainate) separately by SVD (Figure 1A) and identify eigenarrays conserved between the two systems (Figure 1B). This is followed by analysis of biological function using Gene Ontology (GO), and gene cis-regulation using Bayesian networks (BN) and our TRAM database of putative regulatory regions and motifs. These analyses are performed separately for either dataset and then the results for the corresponding eigensystems are compared (GO terms) or statistically cross-validated (BN results) on the other dataset. The cross-validation between the stroke and seizures data is not contradictory with the goal of gaining information by comparison of the two, because the two experimental models can be assumed - on biological grounds - to share some, but not all, regulatory mechanisms. Note that features specific for one model can be identified, as for each model we separately account for the multiplicity of testing.

### Distinct eigengenes following stroke and seizures

**Figure 2:**
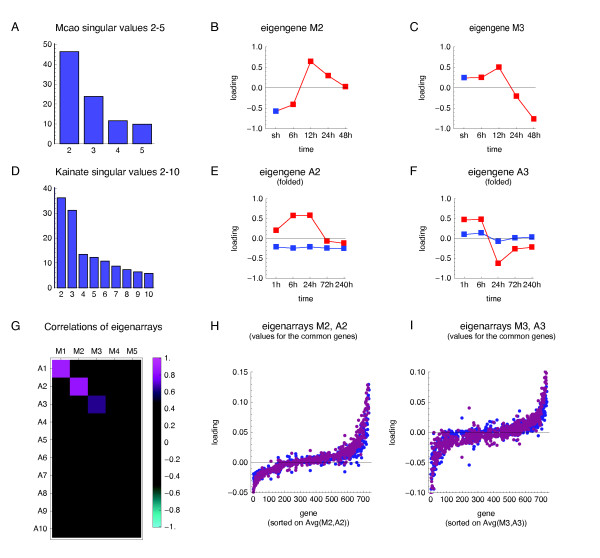
**Comparative SVD analysis of gene expression following ischemia and seizures**. The MCAO and kainate dataset were each separately transformed by SVD and the results were compared. (**A, D**) The singular values plotted as bars. The large singular values for the respective first eigensystems reflecting the magnitude (constant in time) are omitted for clarity. (**B-C, E-F**) The two most important non-constant eigengenes in the MCAO system (M2, M3) and in the kainate system (A2, A3). Red squares indicate loadings on the conditions of treatment, blue - control. The eigengenes A2 and A3, which are vectors of length 10, have been folded in (E-F), to match the loadings onto the same time-points following the injection of kainate and saline. (**G**) Correlations between eigenarrays from either system for the 737 common genes. (**H-I**) Loadings of the respective second (**H**) and third (**I**) eigensystems, in the MCAO (blue) and kainate (violet) model, to the expression profiles of the 737 common genes. The genes were sorted on each gene's average loading of M2 and A2 (**H**) or of M3 and A3 (**I**).

The global temporal changes in gene expression following MCAO in the stroke model are dominated by the top three eigensystems (Figure 2A). The eigengene of the first eigensystem in the MCAO dataset (M1, "M" to indicate MCAO) is constant in time (data not shown) in the log-expression space and thus represents the average level of expression across all the conditions. The second eigengene (M2) represents an increased expression, as compared to control value, at 12-48 h following MCAO, with a peak at 12 h (Figure 2B). The third eigengene (M3) represents a complex pattern with an increase in gene expression at 12 h followed by down-regulation of expression at 24 h and further drop at 48 h (Figure 2C). Notably, the three top eigengenes indicate no changes in gene expression at 6 h after MCAO, which is in agreement with our earlier PCR results showing no changes in mRNA levels of a smaller number of genes [24].

The global temporal pattern of gene expression following kainate-induced seizures in adult rats is dominated by the top three eigensystems (Figure 2D), of which the first again represents the magnitude (data not shown). The second eigengene (A2), represents an increased expression following the injection of kainate; starting at 1 h, largest at 6 and 24 h, returning to the baseline level at 72 and 240 h; and no change at any time-point after the injection of saline (Figure 2E). The third eigengene (A3) represents an increased expression at 1 and 6 h after the injection of kainite; followed by strong decrease in expression at 24 h, continuing, but less pronounced, also at the 72 and 240 h (Figure 2F).

Despite their overall similarity, the corresponding eigengenes are distinct between the two experimental models. In particular, eigengenes M2 and M3 show no change in expression at 6 h following the MCAO, in contrast to eigengenes A2 and A3, showing an increase at 6 h following the injection of kainate.

### Conserved eigenarrays following stroke and seizures

The kainate datasets comprised of expression profiles for 2786 genes (distinct Ensembl gene_stable_id) that significantly changed expression and the stroke dataset consisted of 2392 such genes, with 737 genes common between the two datasets. The correlation analysis revealed that the top three eigenarrays (compared for the common genes) were highly correlated (Figure 2G). The correlations between the respective first, second, and third eigenarrays were 0.87, 0.84, and 0.63, respectively. Note that the correspondence between the three conserved eigenarrays was one-to-one. Given the length (737) of the correlated vectors, these correlations are highly significant (p-values: 10 ^-229^, 10 ^-197^, 10 ^-83^, respectively, assuming independence of genes). This indicates that the top three eigenarrays are highly conserved between the two datasets. Figure 2H-I shows directly genes' loadings of the respective second (H) or third (I) eigensystem in the two datasets, sorted on their average loading in both datasets. This sorting of the genes aids visualization of the eigenarrays conservation, but is not in any way a reason for it, as the correlations shown in Figure 2G were computed before the sorting (and would not be affected by it, anyway). The tangent-like shape of the plots reflects the bell shape of the distributions of genes' loadings of eigensystems 2 and 3.

Further, we focus on eigensystems 2 and 3 characterized by conservation of their eigenarrays occurring despite differences between the corresponding eigengenes (Figure 2B vs. E, C vs. F). This suggests that the two eigensystems reflect regulatory inputs operating in both systems, but with different kinetics and relative strengths.

### Separation of effects of biological processes on gene expression

A universal reason underlying co-regulation of genes is participation of their products in a common biological process. To assess if the contribution of the eigensystems 2 and 3 to the gene expression profiles is associated with biological functions, we analyzed the Gene Ontology "biological process" annotations of all genes in either dataset, ranked on the loadings of the respective eigensystems 2-3.

**Figure 3:**
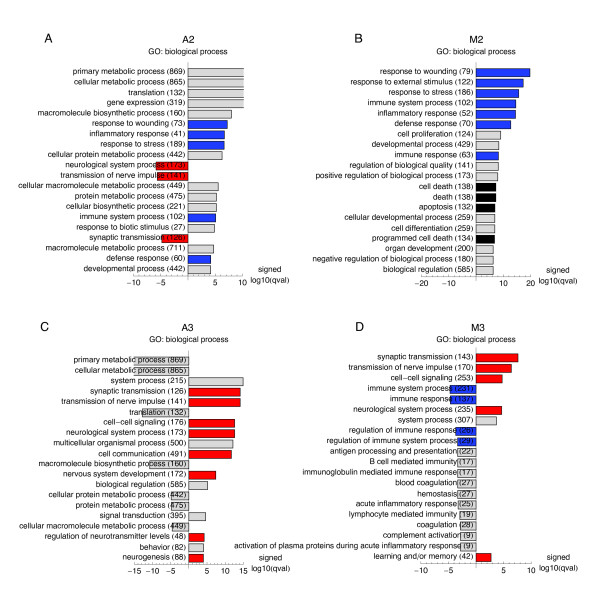
**Functional Gene Ontology annotations associated with the conserved eigensystems **(**A**-**D**) Association of loadings of the conserved eigensystems with the functional annotations from the GO "biological process" ontology were analyzed by Wilcoxon sign rank test using RankGOstat [55]. Twenty GO terms most associated with a given eigensystem, and their association FDR q-values are shown as bar plots. For the plots the q-values were log10-transformed and multiplied by +1 or -1, to reflect association with the positive or negative loadings of a particular eigensystem. GO terms with overlapping meanings (identified by human inspection) are indicated by the same colour of the bars, with red marking terms related to "synaptic transmission", blue marking terms similar to "inflammatory response", and black marking terms describing cell death/apoptosis.

In both experimental models, the positive loading of the second eigensystem was significantly associated with overlapping GO terms describing the inflammatory response to the brain injury (Figure 3A, B). Additionally, in the MCAO system the positive loading of eigensystem M2 was also significantly associated with GO terms describing programmed cell death (apoptosis).

In the kainate system, the positive loading of eigensystem A3 was highly significantly associated with several overlapping GO terms describing neuronal activity, such as: synaptic transmission, transmission of the nerve impulse (Figure 3C). No such association was detected for third eigensystem (M3) from the SVD on the MCAO dataset, following its initial filtering (ANOVA p-value < 0.05). However, when the GO analysis was repeated for the third eigenarray in the SVD result on the MCAO dataset filtered at ANOVA p-value < 0.5 and thus containing more genes, there was a clear association between the loadings of the third eigensystem and GO terms describing neuronal activity (Figure 3D). Loosening of the p-value threshold was possible, because the top three eigensystems were extremely robust to the change of the p-value threshold, with eigenarrays correlations > 0.999 between vectors of length 2786 for the change of the threshold from 0.05 to 0.5 (data not shown). Comparison of the singular values (Figure 2A vs. 2D) indicates that the relative contribution of the conserved third eigensystem (reflecting neuronal activity) was higher in the kainate system, while the relative contribution of the conserved second eigensystem (reflecting inflammation and/or apoptosis) was higher in the stroke.

### Bayesian networks analysis of cis-regulation of the conserved eigensystems

**Figure 4:**
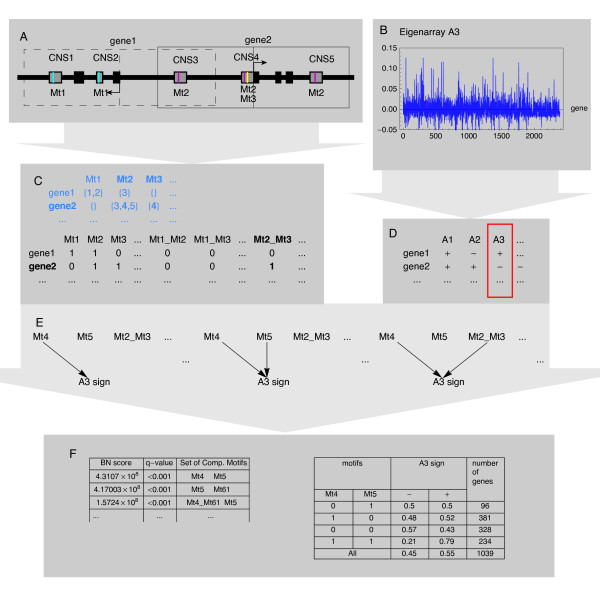
**Bayesian network model of fragmented cis-regulatory regions **(**A**, **C**) Sequence preprocessing consists of extracting instances of composite motifs i.e. sets of (up to three motifs) in the same conserved non-coding sequence (CNS), from the flanks of transcription start sites of all human-rat orthologous genes. (**B, D**) Expression data preprocessing consists of SVD, followed by discretization of expression into up- and down-regulation in the subspace of a particular conserved eigensystem - based on the sign of its loading. (**C, D**) Composite motifs and expression data are combined in one dataset, in which the data records correspond to genes. (**E**) This dataset becomes an input for our Bayesian networks (BN) learning algorithm, which identifies sets of composite motifs most associated with the sign of loadings of a given eigensystem. (**F**) The final output consists of a ranking of such sets, with conditional probability distributions representing their impact on a given eigensystem. BN learning was performed independently for each of the eigensystems: A2, A3, M2, M3; on the data for all the genes in the respective dataset. Eigensystem A3 is shown as an example.

Conservation of eigenarrays suggests that they reflect regulatory mechanisms, possibly operating at the level of transcription regulation. To identify such mechanisms, we employed Bayesian networks, previously successfully applied to modelling transcriptional regulation [14,15,17-20]. We follow the above approaches in general, but several essentials are specific to our methodology:

• Regulation of gene expression is analysed separately for each conserved eigensystem. In the subspace of a given eigensystem gene expression is binarized into up- and down-regulation, according to the sign of its loading. (Figure 4B, D).

• Our combinatorial model of cis-regulation takes into account fragmentation of metazoan cis-regulatory regions into multiple conserved non-coding sequences (CNSs) [25,26], and distinguishes between co-occurrence of several TF-binding motifs in the same CNS and their co-occurrence in the same gene (Figure 4A, C). Following previous work [27,28], we term every possible subset of the motifs present in the same CNS a composite motif.

• Regulatory mechanism is predicted by learning Bayesian networks with an exact algorithm. Computations are performed by double application of the BNFinder program [29]. The first run selects the most promising composite motifs (possibly single motifs), while the second run selects the sets of such composite motifs that best predict the sign of the loading the chosen eigensystem (Figure 4E, F).

**Figure 5:**
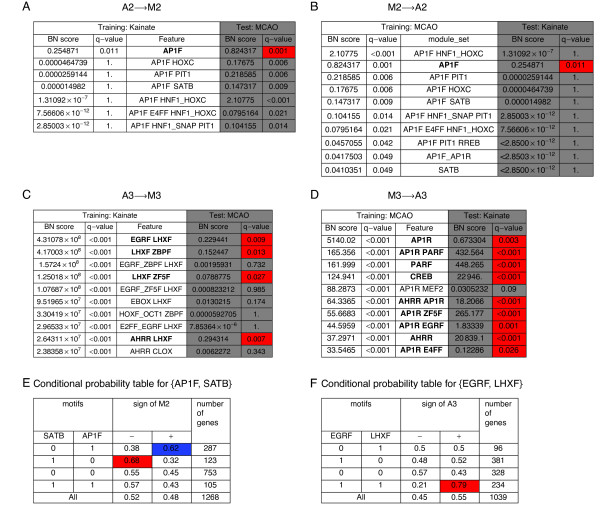
**BN analysis of cis-regulation for the conserved eigensystems**. The four tables (**A-D**) present the results of BN analysis of cis-regulation for the conserved second and third eigensystems from either dataset, followed by testing of highest-ranking features on the corresponding eigensystem from the other dataset. In each panel, the column Feature lists up to 10 nonempty sets of composite motifs with highest BN score and q-value < 0.05 on the indicated training dataset. Note that single motifs are included in the set of composite motifs. BN score of a composite motif set is the ratio of its posterior probability to the posterior probability of the empty set. The corresponding q-value derives from the permutational test. The shaded columns give the values of BN score and the corresponding q-value for the same feature computed on the other (test) dataset. Red color marks the cells with the test q-values < 0.05 for the features that also had training q-value < 0.05 and the descriptions of such features are given in bold. The q-values take into account the multiplicity of testing for each dataset separately, so it is possible to identify the features significant for one dataset only. (**E, F**) The conditional probability tables for the pairs of motifs: {AP1F, SATB} (E) and {EGRF, LHXF} (F).

Four BN analyses were performed, separately for each conserved eigensystem in either experimental model (M2, A2, M3, A3). BN scores were directly converted to q-values - the false discovery rate [30] analogue of p-values, by comparing each feature's score on the original data to the distribution of scores from 1000 BN analyses on permuted data - each following an independent random permutation and assignment of expression values to the genes' putative cis-regulatory regions. The conservation of the two eigensystems between the stroke allowed for selection of best features on one dataset (we choose up to ten features with training q-values < 0.05) and then testing them on the other - containing the data for largely different genes. The training and testing were performed for the conserved second (Figure 5A, B) and third eigensystem (Figure 5C, D) in both directions. During the test we used the same q-values as during the training, i.e. they were corrected for all the hypotheses ever looked at on the test dataset. We note that this is a very stringent correction, as only up to 20 hypotheses were considered for each eigensystem during the test stage (up to ten for either direction of the comparison).

### Antagonistic effects of motifs binding AP1 and SATB on gene expression following the stroke

BN search identified just one feature, namely the motif AP1F - a family of binding sites for the transcription factor AP1 (Additional file 2) as the feature significantly (q-value < 0.05) associated with the positive sign of eigensystem A2 in the kainate model (Figure 5A, columns: "Training: Kainate"). Notably, this feature was significantly associated with the corresponding eigensystem M2, when tested on the dataset from the MCAO model (Figure 5A, column Test: MCAO). The choice of the MCAO data as the training dataset resulted in identification of 7 significant features, of which the second was again AP1F, and only this feature was significant also in the cross-system test on the kainate dataset (Figure 5B, columns "Test: kainate"). All remaining features identified as significant (q-value < 0.05) on the training datasets included AP1F as one motif, and two of them were pairs of AP1F with another motif in the same gene. Of the features significant in the MCAO system, particularly interesting is the pair {AP1F, SATB} - a set of two motifs co-occurring in the same gene, which have antagonistic effects on expression in the subspace of eigensystem M2. The presence of motif AP1F in the absence of SATB in the same gene was associated with the positive sign of M2 loading, while the presence of SATB in the absence of AP1F was associated with the negative M2 loading (Figure 5E).

### Identification of known and new regulators/targets for the eigensystem reflecting synaptic activity

BN search identified a number of features as highly significantly (q-value < 0.001) associated with the sign of M3 loading during the training on the kainate dataset. The ten highest-ranking features, ranked on their BN score were tested on the MCAO dataset (Figure 5C). Of the top ten features significant on the kainate dataset, four were also significant on the MCAO dataset. All of these features, marked in bold in Figure 5C, were pairs of motifs co-occurring in the same gene. All these pairs contained LHXF as one motif, with EGRF, AHRR, ZF5F or ZBPF as the other motif. The highest-ranking feature - the pair EGRF and LHXF in the same gene, but neither motif of its own, was 79% specific for the positive sign of eigensystem 3 (Figure 5F). When the training was performed on the MCAO dataset, several features significantly (q-value < 0.001) associated with the sign of M3 were identified (Figure 5D). Importantly, out of the top ten features identified on the stroke dataset, nine were also significantly associated with the same sign of M3 on the kainate dataset. The features significant in the cross-system test were either single motifs (AP1R, PARF, CREB, AHRR) or pairs of motifs in the same gene. All these pairs contained AP1R as one motif, with PARF, AHRR, ZF5F, EGRF, E4FF as the other motif. Three motifs, namely EGRF, ZF5F, AHRR were common between the top ten features identified during training on the kainate and the MCAO datasets.

### Effects of multiplicity of motifs and CNSs on gene log-expression

We wanted to check if a model taking into account motif multiplicity would allow a more precise prediction of the value of expression. Therefore, we applied a linear regression analysis to the motifs identified by BN analysis as significant in both systems, and additionally the motif SATB significant in the MCAO system only. For the reasons detailed in the Materials and methods, we always performed a weighted linear regression, with the average loadings in groups of genes with the same motif count as the response variable, and the weights set to the numbers of genes in each group, as suggested by Faraway [31].

**Figure 6:**
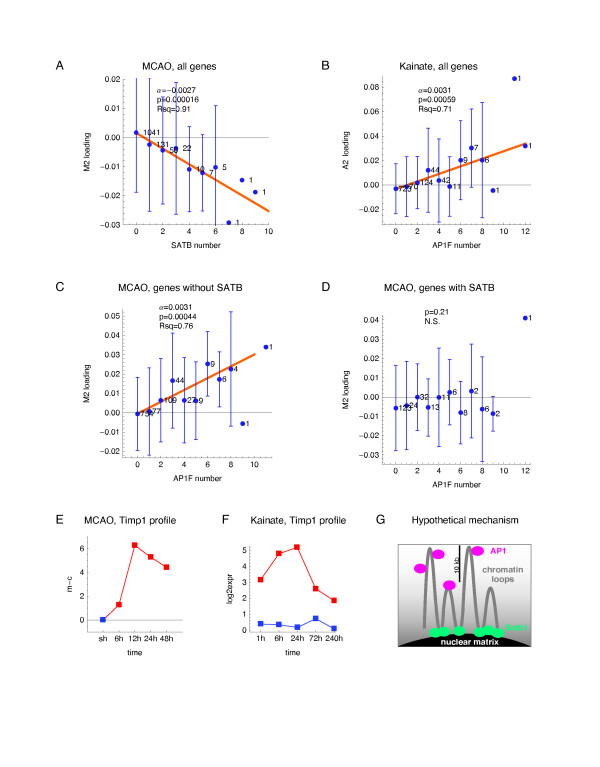
**Effects of motifs binding AP1 and Satb1 on gene expression in the subspace of conserved eigensystem 2**. The effects of motif count per gene on the loadings of the indicated eigensystem were analyzed by weighted linear regression. The response variable was the average loadings of a given eigensystem in groups of genes with the same count of the motif used as the regressor variable, with the weights equal to the numbers of genes per group. The average loadings for each motif count are indicated as blue dots, with their standard deviations shown as error bars, and the group gene count plotted next to each fitted data point. (**A**) The effect of SATB count on the loadings of eigensystem M2. (**B**) The effect of AP1F count on the loadings of eigensystem A2. (**C**) The effect of AP1F count on the loadings of eigensystem M2 analyzed for the genes without SATB motif. (**D**) The effect of AP1F count on the loadings of eigensystem M2 analyzed for the genes with SATB motif. (**E-F**) The log-expression profiles of Timp1 in the MCAO and kainate system. (**G**) A hypothetical mechanism, by which binding to the nuclear matrix via Satb1 makes a gene less accessible for binding or activation by AP1.

The regression analyses were performed separately for the MCAO and the kainate datasets. The linear regression confirmed that the AP1F and SATB motifs had antagonistic effects on expression in the subspace of eigensystem M2 (Figure 6A-C). The count of motif SATB per gene had a clear linear (R ^2 ^= 0.91) and highly significant (p = 1.6 × 10 ^-5^) effect on the group-average expression in the subspace of eigensystem M2 (Figure 6A). In agreement with the earlier BN result, the count SATB had no effect on loading of eigensystem A2 (data not shown). The inhibitory effect of SATB on gene expression in the MCAO system was specific for eigensystem M2, with no inhibition of expression in the subspace of any other eigensystem (data not shown).

The count of motif AP1F had a significant, positive and possibly linear effect on the average expression in a subspace of the second eigensystem, both in the MCAO (p = 0.0019, R ^2 ^= 0.64) and in the kainate dataset (Figure 6B, p = 0.00059, R ^2 ^= 0.71). Remarkably, when the effect of AP1F count on M2 loading was analyzed separately for the genes with and without motif SATB, the effect became more apparent for the genes without motif SATB (Figure 6C, p = 0.00044, R ^2 ^= 0.76), while the effect was nullified for the genes with the motif SATB (Figure 6D).

**Figure 7:**
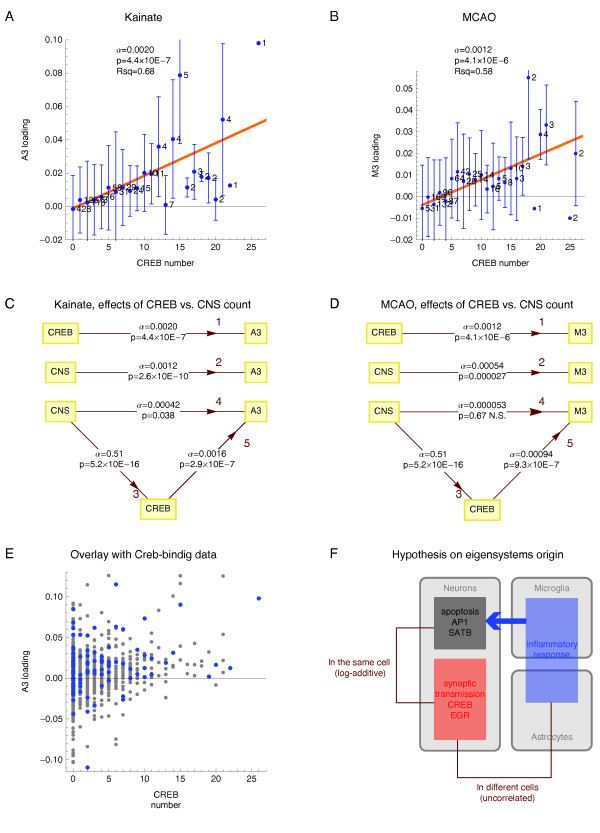
**Effects of CREB motif count on gene expression in the subspace of the conserved eigensystem 3 **(**A, B**) The effects of CREB motif count per gene on the average loadings of the eigensystem A3 or M3 analyzed by weighted linear regression, as described in the legend to Figure 6. (**C**, **D**) Effect of CREB count and direct and indirect (via CREB count) effect of CNS count per gene on the average loadings of eigensystems A3 or M3 analyzed by weighted linear regression, either univariate (edges 1, 2, 3) or bivariate (edges 4, 5), in groups of genes with the same numbers of CNSs, CREB motifs, or both. The results are represented as path analysis graphs, with each edge marked by the values of the respective linear regression directional coefficient α and its corresponding t-test p-value. In the univariate regression of CREB count on CNS count (edge 3) the data for all the genes with at least one CNS in the TRAM database were used. (**E**) The single gene A3 loadings and CREB counts for all the genes with CNS(s) in the kainate dataset (grey dots) compared to the values the Creb-binding genes in PC12 cells identified by genome-wide ChIP analysis by Impey et al. [50] (blue dots). (**F**) Uncorrelated, additive effects of the motifs SATB and CREB on gene log-expression provide an insight into the biology of the MCAO system.

The linear regression revealed that the count of motif CREB had a highly significant and approximately linear effect on the average expression in a subspace of the third eigensystem in the kainate (Figure 7A, p = 4.4 × 10 ^-7^, R ^2 ^= 0.68) and in the MCAO system (Figure 7C p = 4.1 × 10 ^-6^, R ^2 ^= 0.58). The effect of CREB sites number on gene expression was specific for the third eigensystem, in particular in the MCAO model, where it had no effect on the loadings of the eigensystem M2 (data not shown).

The effects of motif multiplicity on gene expression prompted us to investigate by the linear regression if a related variable - the count of conserved non-coding sequences (CNSs) per gene had an effect on gene expression. That we found was true in both experimental models (Figure 7E, F). Similarly to the effect of CREB count, the effect of CNS count was highly specific for the third eigensystem (data not shown). However, when the effect of CNS count was analyzed in a bivariate linear regression model, together with that of CREB, the effect of the CNS was completely (MCAO) or nearly completely (kainate) dependent on the CNSs' content of Creb-binding motifs (Figure 7E, F).

## Discussion

Here, we demonstrated that eigensystems conserved between stroke and seizures separate effects of inflammation/apoptosis and synaptic activity on gene expression. The contribution of the eigensystem 3 reflecting synaptic activity was relatively greater (compared to eigensystem 2) in the seizures model, in agreement with higher electrical activity of neurons. Remarkably, our analysis of cis-regulation revealed that the these two functionally well-interpretable eigensystems were regulated by distinct sets of transcription factors, with AP1 and SATB regulating the eigensystem reflecting inflammation/apoptosis, and numerous TFs including Creb and Egr regulating the eigensystem reflecting neuronal synaptic activity.

Activation of transcription factor AP1 following the kainate-induced seizures and cerebral ischemia is well established [32,33]. In particular, Timp1 was shown to be the target of AP1 following kainate-induced seizures [34]. The mRNA profiles of Timp1 in both systems (Figure 6E, F) are highly similar to the profiles of the respective second eigengenes, which is compatible with our identification of AP1 as the key regulator of this eigensystem. It is well established that activation of Mapk8-Jun/AP1 signalling pathway has a predominantly pro-apoptotic effect in neurons [35], however, only few Mapk8-AP1 targets genes have been identified. Therefore, demonstrating the importance of the number of AP1-binding motifs per gene and the simultaneous absence of SATB motif for gene activation contributes to identification of AP1 target genes.

We report novel and exciting finding that presence of the motif binding Satb1 prevents - in a motif number dependent manner - transcriptional activation in the stroke system. Satb1, which is the best characterized MAR-binding protein, has recently emerged as a key factor integrating higher-order chromatin architecture and gene regulation - reviewed in [36]. Depending on cell type and locus, its effect on chromatin looping may either activate transcription, as described for Th2 interleukin gene cluster [37], or inhibit transcription, as for the MHC class 1 locus [38] and tentatively for our eigensystem M2. A hypothetical mechanism, in which genes in longer chromatin loops, or at the peaks of the loops, are more accessible to binding or activation by AP1, is depicted in Figure 6G. Proteolytic degradation of Satb1 occurs during early phases of apoptosis [39-41]. In the current work, the effect of SATB motif on expression was limited to the MCAO eigensystem 2 associated with the apoptosis.

Our analysis of cis-regulation of conserved eigensystem 3 - reflecting neuronal (synaptic) activity correctly predicted the known role of Creb/Atf/E4f1 and Egr as key regulators of neuronal activity regulated genes, important for neuronal plasticity and memory - for review, see [42,43]. CREB motif binds transcription factors of the Creb family [43-45], while E4FF motif binds transcription factors from the Atf family. EGRF binds transcription factors of the Egr family [46,47]. PARF binds PAR/bZIP family of TFs (Dbp, Hlf, Tef, and Vbp1). The motifs binding Creb, Atf and Vbp1 are similar (Additional file 2) and these transcription factors have been shown to bind to overlapping sites [48]. A loss of the PAR/bZIP transcription factors results in seizures [49]. Using classical experimental methods, about a hundred Creb target genes have been identified, of which about half encodes neuron-specific proteins - reviewed by Lonze & Ginty [44]. A genome-wide chromatin immunoprecipitation study by Impey et al. identified Creb binding genes in the neuron-like differentiating rat pheochromocytoma PC12 cells [50]. When this set of genes was analyzed in our datasets, we found a clear association between Creb-binding to the gene and the positive loading of the third eigensystem (Figure 7D). Thus, the experimental data of Impey and co-authors support our in silico results, demonstrating an importance of the presence of CREB motif for gene up-regulation in the subspace of eigensystem reflecting neuronal activity.

Much experimental evidence supports an important role of Egr transcription factors in brain function. Transcription factors from the Egr family are induced in the rat hippocampus following kainate-induced seizures with kinetics closely resembling eigengene A3 (data not shown) and regulate expression of Arc [51], a gene important for neuronal plasticity and memory formation [52]. Transcriptional activation of Egrs was also demonstrated following brain ischemia - reviewed in [47]. In addition to Creb and Egr, our BN analysis identified several novel tentative transcriptional regulators of the eigensystem reflecting synaptic activity (Figure 5 and Additional file 2).

We demonstrate linear effects of the counts of the motifs SATB and CREB on log-expression in subspaces of the respective regulated eigensystems following the MCAO. These findings are in agreement with the predictions of the linear response model of gene regulation [21]. Moreover - because this model is valid only for TFs operating within the same cell - the observed agreement is revealing of the underlying biology (Figure 7F). First, it suggests that Satb and Creb operate within the same cells, namely neurons. This prediction is in agreement with our previous experimental data that majority of the cells undergoing apoptosis in the MCAO system are neurons [24]. Second, our results suggest that neuronal apoptosis is triggered by inflammation occurring in other cell types, namely microglia and astrocytes. This could explain why effects of inflammation and apoptosis are reflected by the same eigensystem, uncorrelated to the one reflecting effects of synaptic transmission.

The observed linear effect of CNSs' count per gene on log-transformed gene expression, depending on their content of CREB, is very interesting in the context of high specificity of this effect (data not shown) for the conserved eigensystem reflecting neuronal synaptic activity. Lee et al. [53] reported relatively greater cumulative length of CNSs in the upstream regions of genes involved in development, cell communication, neural functions and signaling processes, and suggested that this may reflect their greater regulatory complexity. We suggest, as another possibility, that neuronal genes need more CNSs (putative enhancers) to accommodate CREB motifs needed for responsiveness to rapidly changing synaptic activity.

Our results, demonstrating conservation of eigenarrays of temporal log-expression profiles, between hippocampus following seizures and cortex following the stroke, corroborate and extend recent findings of Oldham et al. [54]. These authors applied SVD to clusters ('modules' in their terminology) of expression profiles identified separately for several brain regions, and demonstrated conservation of 'module membership' between the corresponding clusters from different regions. As the 'module membership' is closely related to the first eigenarray of each cluster, their findings imply conservation of the first eigenarrays between the corresponding clusters. Our results demonstrate conservation of eigenarrays that occurs genome-wide for three eigensystems, two of which reflect distinct well-defined biological processes and are regulated via different sets of transcription factor binding sites.

## Conclusions

Eigensystems conserved between stroke and seizures separate effects of different biological processes on gene expression, exerted via distinct sets of transcription factor binding motifs. Motif recognized by the nuclear matrix attachment region-binding protein Satb1 blocks AP1-driven transcriptional activation. The effects of motifs binding Creb and Satb1 on gene expression conform to the assumptions of the linear response model of gene regulation.

## Methods

### Gene expression profiling in the MCAO system

#### Animals and surgical procedures

The experimental protocol was approved by the Local Animal Care and Use Committee and conforms to the national guidelines for the care and use of animals in research. 3-months old male Wistar rats weighing 270-320 g were used.

The MCAO (a middle cerebral artery occlusion) surgeries were performed under general halothane anaesthesia. Transient MCAO was induced with the intraluminal filament method (3-0 nylon monofilament suture) as described before [24]. A filament was withdrawn after 90 min. of MCAO to allow reperfusion, the incision was closed and anaesthesia discontinued. Sham-operated animals were subjected to the similar surgery with exception of MCA occlusion.

#### RNA isolation and microarray hybridization

At various times after reperfusion, sham-operated and MCAO subjected rats were anesthetized with an overdose of pentobarbital and decapitated. Brains were rapidly removed, bisected at the midline and dorsolateral fragments of cerebral cortex containing MCA territory was dissected from the ipsilateral to occlusion (right) and contralateral (left) hemisphere. Total RNA was extracted from the samples using a phenol-guanidine thiocyanate-based method (TRI REAGENT, Sigma, Germany) and cleaned using RNeasy Total RNA kit (Qiagen, Germany) according to the manufacturer's recommendations followed by DNAse treatment. The amount and quality of the RNA was determined by spectrophotometry and capillary electrophoresis. The microarray hybridizations were conducted in the microarray facility of the Institute of Oncology, Maria Sklodowska-Curie Memorial Cancer Center, Gliwice Branch, Gliwice, Poland. Each time-point (6, 12, 24, 48 h) and sham-operated (sh) group consisted three animals per group; RNAs from each individual were separately labelled and analyzed by microarray hybridization, for a total of 15 microarray hybridizations. The experiment was loaded to ArrayExpress (accession E-MEXP-2222).

### Source of the kainate gene expression data

The published dataset of Wilson et al. [23] from expression profiling in the hippocampus of adult rats with Affymetrix RG-U34A chip was downloaded from the NIH Neuroscience Microarray Consortium http://arrayconsortium.tgen.org/, projects: Koh-7K08NS002068-05-3, Koh-2K08NS002068-04. These datasets probed gene expression in the hippocampus of adult (P30) and young (P15) rats at 5 time-points (1, 6, 24, 72, 240 h) following the intraperitoneal injection of kainate (treatment) or saline (control). Only animals with nearly continuous seizures for more than half an hour were included in that study. Age-specific doses of kainate (3 mg/kg at P15, and 10 mg/kg at P30) were used that had been determined previously to result in < 25% mortality while inducing seizures in >60% of the animals. At the time of RNA isolation the animal could be seizing or during the latent period. Each condition was probed by three microarray hybridizations. The kainate data from both projects were pre-processed together, and the MAS5 detection calls for both ages were used together for the P/A/M filtering described below. Subsequently, the Mas5 signal data only from the adult rats (10 conditions) were used in the current work.

### Pre-processing and annotation of the expression data

The CEL files from the MCAO experiment and separately the CEL files from the kainate experiment (from the young and adult rats together) were pre-processed with the MAS 5.0 algorithm as implemented in the affy R Bioconductor package (Irizarry et al. 2002). Only the profiles of the probesets detected (MAS 5 call: Present or Marginal) in all hybridizations for at least one condition in a given experiment were used. The profiles from either experiment identified by probe set identifiers were mapped to the Ensembl 39 gene_stable_ids. Separately for either dataset, we computed a single average MAS5 signal profile for each gene_stable_id, resulting in gene expression matrices: (11012 × 15) for the MCAO system, and (3908 × 30) for the ***A***dult rats from the kainate system. These data matrices were log2 transformed and analyzed separately by ANOVA. For further analysis from either dataset we selected the genes with the respective ANOVA p-value < 0.05. The average log2 expression profiles of these genes over the three biological replicates were computed, resulting in matrices: *M *(2786 × 5) for the MCAO system, and *A *(2392 × 10) for the kainate system.

### Comparative SVD analysis

The SVD analysis and the comparison of eigenarrays between two datasets were performed essentially as previously described [11]. Briefly, SVD was performed separately on matrices *M*, *A*, resulting in matrices *u *_*M *_(2786 × 5), *m *_*M*_, *v *_*M *_and *u *_*A *_(2392 × 10), *m *_*A*_, *v *_*A *_respectively.

For the comparison of loadings between the MCAO and kainate dataset, from the matrices *u *_*M *_and *u *_*A *_we selected the rows (gene loadings vectors) for the 737 genes common between these two datasets. This resulted in matrices *u *_*MA *_and *u *_*AM*_. We calculated the Pearson correlation coefficient *r *between each pair of columns of *u *_*MA *_and *u *_*AM*_. The two-sided *p*-values corresponding to these correlations were obtained from the Student *t *distribution, with the *t *statistics calculated with the formula *t *= *r*[*d */(1-*r *^2^)] ^1/2^, where *d *is the number of the degrees of freedom.

### Gene Ontology annotation

GO terms associated with loadings of conserved eigensystems were identified, separately for either dataset, using RankGOstat [55], available at http://gostat.wehi.edu.au/. The lists of gene symbols (Ensembl display_id), together with loadings of a particular eigensystem for a given (ANOVA-filtered) dataset were used as the input files. Default options (Wilcoxon Signed Rank test, Benjamini False Discovery Rate correction for multiple testing) were used, with the RGD database chosen as the source of GO annotations and the analysis was restricted to the "biological process" ontology. The result files were saved, parsed and converted to graphics using local scripts.

### Transcription regulatory regions and motifs (TRAM) database

#### Putative regulatory regions

We used conserved non-coding sequences (CNSs) between human and rat as putative regulatory regions. For each human-rat orthologous gene pair (ortholog_one2one and apparent_ortholog_one2one) in Ensembl release 39, a flank of 20 kilobase (kb) of the genomic sequence from -10 kb to + 10 kb from the transcription start site were aligned using the AVID global alignment algorithm [25]. Sequence windows at least 100 base-pairs (bp) long with ≥ 75% identity were selected as putative regulatory regions. This resulted in the identification of 49425 CNSs for 9099 orthologous gene pairs in the human and rat genomes. A large proportion of similarly identified human-rodent CNSs was shown experimentally to function as enhancers [26]. The input genomic sequence and annotation data, and the results of this analysis were stored in a relational database named TRAM (**T**ranscription regulatory **R**egions **A**nd **M**otifs), built on the open MySQL platform. The average length of the CNSs was 190 +/- SD 136 bp.

#### Motifs and composite motifs

Instances of transcription factor binding motifs were predicted for all the vertebrate nucleotide distribution matrices of the Matrix Family Library version 6.2 using the program MatInspector [56] (Genomatix). Default thresholds, optimized for each motif as described in [57] were used. Search was performed for all CNSs in the TRAM database, separately for the human and the rat sequence, resulting in identification of 1679998 vertebrate motif instances in the human and 1601216 in the rat. The motif library contained 464 vertebrate nucleotide distribution matrices grouped into 151 matrix families [57]. Motifs identified with matrices from the same family were treated as the same non-redundant (n-r) motif identified by the family name. An instance of a n-r motif X in a given CNS is defined as conserved, if both the human and the rat sequence of this CNS contain at least one instance of X (not necessarily in the same AVID-aligned position). According to this definition, TRAM contains 1061884 instances of conserved n-r motifs. Only the conserved n-r motifs, referred to as "motifs" in the main text, were used in further analysis.

A composite motif X_Y_... is defined to have an instance in a CNS if this CNS contains at least one instance of each of the conserved n-r motifs X, Y, ... . Note that every single motif is also a composite motif.

### Bayesian networks analysis

In our model of transcription regulation the set of Bayesian network vertices is split into two subsets: cis-regulatory features (composite motifs) and expression patterns (sign of the loading of a particular eigensystem). Furthermore, all the edges lead from cis-regulatory features to a particular expression pattern. In order to identify these relationships, we learn Bayesian networks from a dataset joining cis-regulatory and expression data for each gene. The input dataset joins presence or absence of every composite motif with the sign of loading of a single conserved eigensystem (Figure 4C, D).

In Step 1 of our procedure (not illustrated) over 100 promising composite motifs (built of up to three motifs) associated with the sign of the chosen eigensystem are identified. Only these selected composite motifs are then used as the input for the Step 2 (Figure 4E) identifying the best sets of composite motifs and their conditional probability distributions (Figure 4F). Each set of composite motifs has a q-value derived from 1000 random permutations of gene labels. For each permutation we created a new cis-regulatory dataset (with gene labels permuted accordingly) and learned the optimal composite motif set. Both steps of a learning procedure were performed with the BNFinder software [29] - a Python package for learning Bayesian networks from data. BNFinder implements the polynomial time learning algorithm dedicated to dynamic Bayesian networks, as well as to static ones with constraints forcing the network acyclicity [58], as is the case here.

We used the Bayesian-Dirichlet equivalence (BDe) [59,60] criterion with priors on the conditional probability distributions according to [59]. A prior on the network structures is proportional to the product of penalty parameters over all the edges in the graph of the refined model. Furthermore, penalty parameters increase with composite motif size. This choice results in a preference for sparse graphs, and thus protects our procedure from overfitting. BN score of a composite motif set was computed as the ratio of its posterior probability to the posterior probability of the empty set. To permit the cross-system validation of BN scores, the sets of composite motifs selected during Step 1 for the corresponding eigensystems (e.g. A2-M2) from either dataset were combined to form their union, which was then used during Step 2.

### Regression analysis

The motif count per gene was defined as the number of instances of conserved non-redundant motifs in the rat sequences of all the CNSs assigned to this gene. Only the genes with at least one CNS were used in the univariate regression analysis when the count of a particular motif was used as the regressor variable. When the CNS count, or CNS count and the motif count, were used as the regressor variable(s), the genes with zero CNS count were also included during the analysis

The single gene loadings of eigensystems 2, 3 were not normally distributed, which precludes statistical interpretation of the results of the regression with single-gene loadings used as the response variable. Therefore, in linear regression analysis, we decided to use the average loadings of a particular eigensystem in groups of genes with the same motif count as the response variable. In the regression analysis on the average values we confirmed the approximate normality of the residua (Additional file 3). Since the average values for different motif counts were computed from different numbers of observations, generally decreasing with the motif count, which was accompanied by changing variance of the loadings, we employed the Goldfeld-Quandt (GQ) test to detect the existence and magnitude of heteroskedasticity. Results of this test indicate that (i) for MCAO data heretoscedastic errors were detected (p < 0.05) in all the regression models. Therefore, we used weighted least squares approach, with weights set to the number of genes in each group [31], which is the well-known solution to the heteroskedasticity problem. The regression analysis was performed in Mathematica 7, and the GQ tests were performed in R.

### Availability of TRAM database and the software

The TRAM database is available from the authors m.dabrowski@nencki.gov.pl as mysqldump.gzip file. BNFinder source code is available for download at http://bioputer.mimuw.edu.pl/software/bnf/. Additional Pyton scripts, linking BNFinder to the TRAM database, are available from the authors dojer@mimuw.edu.pl.

## Authors' contributions

MD conceived of the study, performed cross-system comparisons, and drafted the manuscript. ND performed the Bayesian Networks analysis. MZ conducted the MCAO experiment. JM performed the linear regression. BK provided biological interpretation and edited the manuscript. All authors participated in writing and approved of the final manuscript.

## Supplementary Material

Additional file 1 **The nomenclature of SVD applied to gene expression data**. Click here for file

Additional file 2 **Sequence logos and transcription factors binding to the motifs identified by BN analysis**. Click here for file

Additional file 3 **Assessment of the normality of the linear regression residua**. Click here for file
