# Supplementary material for: Comparative analysis of cis-regulation following stroke and seizures in subspaces of conserved eigensystems
Source: BMC Syst Biol. 2010 Jun 17;4:86. doi: 10.1186/1752-0509-4-86 (PMC2902439; doi:10.1186/1752-0509-4-86)
Supplement: Additional file 1 — The nomenclature of SVD applied to gene expression data. [file 1752-0509-4-86-S1.PDF]

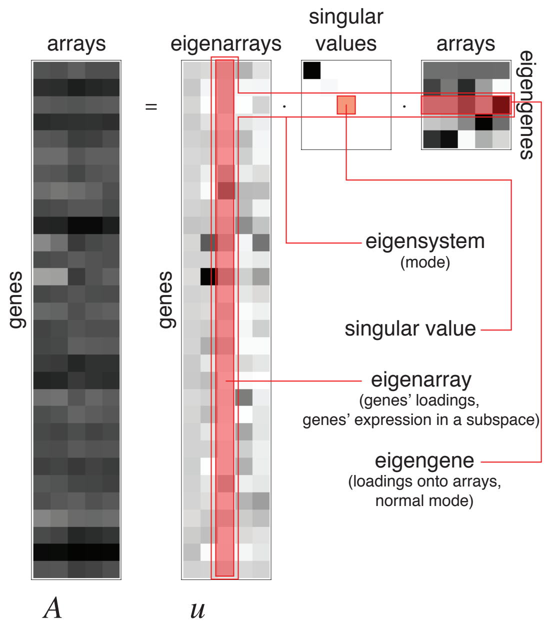

Additional file 1 - The nomenclature of SVD applied to gene expression data

The rows of the matrix *A* are the expression profiles of individual genes across the time-points. These row expression vectors are referred to as “genes”. The columns of the matrix *A* list the expression of all genes at a particular time-points. These column expression vectors are referred to as “arrays”. The singular value decomposition (SVD) transforms the expression matrix *A* from the original basis of genes and arrays to the bi-orthogonal basis of eigengenes and eigenarrays. In this new basis, expression of each gene becomes a weighted sum of contribution of all eigengenes, with loadings of the consecutive eigengenes for this gene contained in the row of the matrix *u*. Each eigenarray lists the loadings of the corresponding eigensystem to the expression profiles of all the genes. The eigenarray, singular value and eigengene collectively constitute an eigensystem. Some of the alternative names used are also given. If before SVD the matrix A is column-centred (each column on its mean value), then SVD corresponds to the principal component analysis (PCA). Then the eigengenes correspond to the principal directions and the eigenarrays to the principal components. Note that the column centring was not performed in the current work.
