# Supplementary material for: Comparative analysis of cis-regulation following stroke and seizures in subspaces of conserved eigensystems
Source: BMC Syst Biol. 2010 Jun 17;4:86. doi: 10.1186/1752-0509-4-86 (PMC2902439; doi:10.1186/1752-0509-4-86)
Supplement: Additional file 2 — Sequence logos and transcription factors binding to the motifs identified by BN analysis. [file 1752-0509-4-86-S2.pdf]

Motif (Matrix Family)	A representative matrix and its logo	Representative transcription factor(s)	Relevant known role of the transcription factor(s)
AP1F	AP1.01 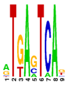	AP1	neuronal death
SATB	SATB1.01 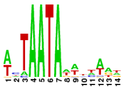	Satb1	attachment to nuclear matrix
EGRF	EGR1.01 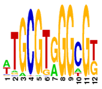	Egr1–3	neuronal activity, neuronal plasticity
ZF5F	ZF5.01 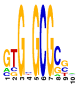	Zfp161	represses FMR1 gene – neuronal plasticity
ZBPF	ZBP89.01 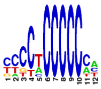	Zbp89	–––
CREB	CREB1.01 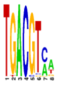	Creb	neuronal activity, neuronal plasticity
PARF	VBP.01 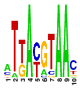	DBP	binding sites overlap CREB, epilepsy, neuronal plasticity
E4FF	E4F.01 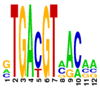	E4f1	binding sites overlap CREB, cell cycle
LHXF	LHX1.01 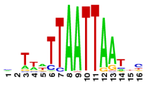	Lhx1–3	motor neuron identity
AHRR	AHR.01 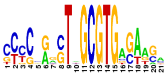	Arnt2	response to hypoxia
AP1R	NFE2.01 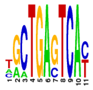	Nfe2l1–2	oxidative stress

Additional file 2 - Sequence logos and transcription factors binding to the motifs identified by BN analysis

For each identified motif (non-redundant motif – see Materials and methods), the name and the sequence logo of a representative nucleotide distribution matrix (Genomatix) are shown, followed by the name of a transcription factor (TF) binding to this motif and relevant known functions of this TF.
