# Supplementary material for: Comparative analysis of cis-regulation following stroke and seizures in subspaces of conserved eigensystems
Source: BMC Syst Biol. 2010 Jun 17;4:86. doi: 10.1186/1752-0509-4-86 (PMC2902439; doi:10.1186/1752-0509-4-86)
Supplement: Additional file 3 — Assessment of the normality of the linear regression residua. [file 1752-0509-4-86-S3.PDF]

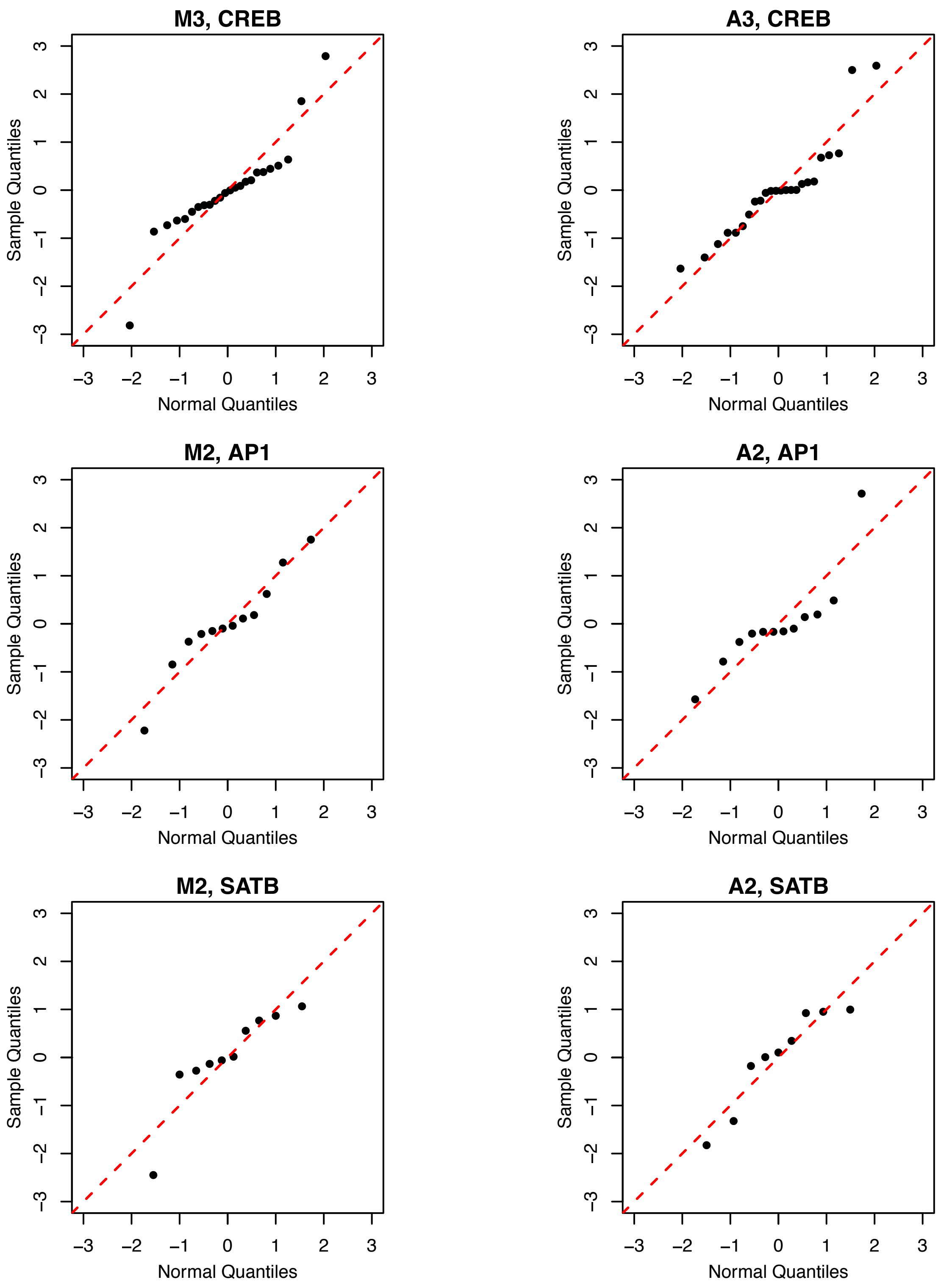

Additional file 3 - Assessment of the normality of the linear regression residua

Quantile-quantile plots of the residua from the univariate weighted linear regression analysis, with the average loadings of the indicated eigensystem in groups of genes with the same count of a given motif used as the response variable and the indicated motif count used as the regressor variable. The weights were set to the numbers of genes in each group. The response and regressor variables are given above each panel.
